# Kardiale Kanalopathien im Kontext hereditärer Arrhythmiesyndrome

**DOI:** 10.1007/s00108-024-01751-x

**Published:** 2024-07-08

**Authors:** Moritz T. Huttelmaier, Thomas H. Fischer

**Affiliations:** Medizinische Klinik 1, Universitätsklinikum Würzburg, Oberdürrbacher Str. 6, 97080 Würzburg, Deutschland

**Keywords:** Long-QT-Syndrom, Short-QT-Syndrom, Brugada-Syndrom, Katecholaminerge polymorphe ventrikuläre Tachykardie, Plötzlicher Herztod, Long QT syndrome, Short QT syndrome, Brugada syndrome, Catecholaminergic polymorphic ventricular tachycardia, Sudden cardiac death

## Abstract

Hereditäre Arrhythmiesyndrome sind seltene Erkrankungen, die allerdings im Kindes‑, Jugend- und jungen Erwachsenenalter eine häufige Ursache des plötzlichen Herztods darstellen. Grundsätzlich kann im Kontext genetischer Erkrankungen eine Unterscheidung zwischen Kanalopathien und Kardiomyopathien getroffen werden. Schwerpunkt der vorliegenden Arbeit sind die Kanalopathien Long- und Short-QT-Syndrom, Brugada-Syndrom sowie die katecholaminerge polymorphe ventrikuläre Tachykardie (CPVT). Eine frühzeitige Diagnose dieser Erkrankungen ist unerlässlich, lassen sich doch durch die medikamentöse Therapie, die Aufklärung über Verhaltensmaßnahmen und gegebenenfalls die Implantation eines Kardioverter-Defibrillators die Prognose und Lebensqualität der Patienten signifikant verbessern. Der Beitrag beleuchtet die pathophysiologischen und genetischen Grundlagen dieser Kanalopathien, beschreibt deren klinische Manifestation und kommentiert die Grundlagen für Diagnose, Risikostratifikation und Therapie.

## Abstract (en)

Genetic arrhythmia disorders are rare diseases; however, they are a common cause of sudden cardiac death in children, adolescents, and young adults. In principle, a distinction can be made between channelopathies and cardiomyopathies in the context of genetic diseases. This paper focuses on the channelopathies long and short QT syndrome, Brugada syndrome, and catecholaminergic polymorphic ventricular tachycardia (CPVT). Early diagnosis of these diseases is essential, as drug therapy, behavioral measures, and if necessary, implantation of a cardioverter defibrillator can significantly improve the prognosis and quality of life of patients. This paper highlights the pathophysiological and genetic basis of these channelopathies, describes their clinical manifestations, and comments on the principles of diagnosis, risk stratification and therapy.

Hereditäre Arrhythmiesyndrome sind allgemein seltene Erkrankungen, die allerdings im Kindes‑, Jugend- und jungen Erwachsenenalter eine häufige Ursache des plötzlichen Herztods darstellen [1]. Kanalopathien und Kardiomyopathien zu unterscheiden, ist von zentraler Bedeutung für das Verständnis hereditärer Arrhythmiesyndrome. Durch Beeinträchtigung der normalen elektrischen oder mechanischen Eigenschaften des Herzens prädisponieren beide Gruppen zu Herzrhythmusstörungen und plötzlichem Herztod.

Kanalopathien sind durch Funktionsstörungen von Ionenkanälen gekennzeichnet

Kanalopathien sind als „elektrische Erkrankungen“ durch Funktionsstörungen von Ionenkanälen gekennzeichnet, die zu einem veränderten kardialen Aktionspotenzial bzw. zu einer Veränderung der intrazellulären Ionenhomöostase führen. Kardiomyopathien als „strukturelle Erkrankungen“ sind hingegen durch Veränderungen definiert, die überwiegend im Bereich der aktiven Myofilamente oder anderer Strukturproteine liegen. Im Folgenden soll der aktuelle Wissensstand zu den kardialen Kanalopathien Long- und Short-QT-Syndrom, Brugada-Syndrom (BrS) sowie katecholaminerge polymorphe ventrikuläre Tachykardie (CPVT) dargelegt werden (Tab. 1). Schwerpunkte sind die jeweilige Pathophysiologie, klinische Manifestation und Diagnosekriterien sowie Strategien zur Risikostratifikation und Therapie.

**Tab. 1:** Übersicht zu Kanalopathien

	Ursächliche Mutation	Diagnosekriterien	Arrhythmie-Trigger	Medikamentöse Therapie und Allgemeinmaßnahmen	ICD-Therapie
*Long-QT-Syndrom*	KCNQ1 (LQT1) KCNH2 (LQT2) SCN5A („gain“, LQT3)	1) QTc > 480 ms oder 2) LQTS-Risiko-Score > 3 3) Nachweis definierender Mutation 4) QTc 460–480 ms + Synkope	Körperliche Belastung (LQT1) Erschrecken, akustischer Reiz (LQT2) Nachtschlaf (LQT3)	1) Betablocker 2) Mexiletin (LQT3)	1) Zustand nach SCD 2) Zustand nach Synkope/VT trotz medikamentöser Therapie
*Short-QT-Syndrom*	I_Kr_, I_Ks_, I_K1_ („gain“) I_CA,L_ („loss“)	1) QTc < 320 ms oder 2) QTc < 360 ms + Zustand nach SCD, Nachweis Mutation oder positive FA	–	Hydrochinidin	1) Zustand nach SCD 2) Zustand nach VT
*Brugada-Syndrom*	SCN5A („loss“)	1) Spontanes Typ-1-EKG 2) Induziertes Typ-1-EKG + Zustand nach SCD, Synkope, positive FA oder SCD bei Verwandten ersten Grades < 45 Jahren	Hoher Vagotonus, Schlaf, Ruhe	1) Vermeidung von schweren Mahlzeiten und Alkoholkonsum 2) Fiebersenkung 3) Vermeidung toxischer Medikamente (https://www.brugadadrugs.org) 4) β-Sympathomimetika oder Hydrochinidin bei VT-Sturm	1) Zustand nach SCD/VT 2) Spontanes Typ-1-EKG und Zustand nach Synkope
*CPVT*	Ryanodin-Rezeptor 2 (RyR2)	1) Bidirektionale VT unter Belastung 2) Nachweis definierender Mutation	Körperliche Belastung	1) Vermeidung stärkerer körperlicher Belastung 2) Betablocker 3) Flecainid	Kritische Indikationsstellung

*CPVT* katecholaminerge polymorphe ventrikuläre Tachykardie, *EKG* Elektrokardiogramm, *FA* Familienanamnese, *„gain“* pathologische Funktionssteigerung, *ICD* implantierbarer Kardioverter-Defibrillator, *„loss“* pathologische Funktionsminderung, *LQT1, 2, 3* Long-QT-Syndrom Typ 1, 2, 3, *LQTS* Long-QT-Syndrom, *SCD* „sudden cardiac death“ (plötzlicher Herztod), *VT* ventrikuläre Tachykardie

## Aktionspotenzial und elektromechanische Kopplung

**Abb. 1:**
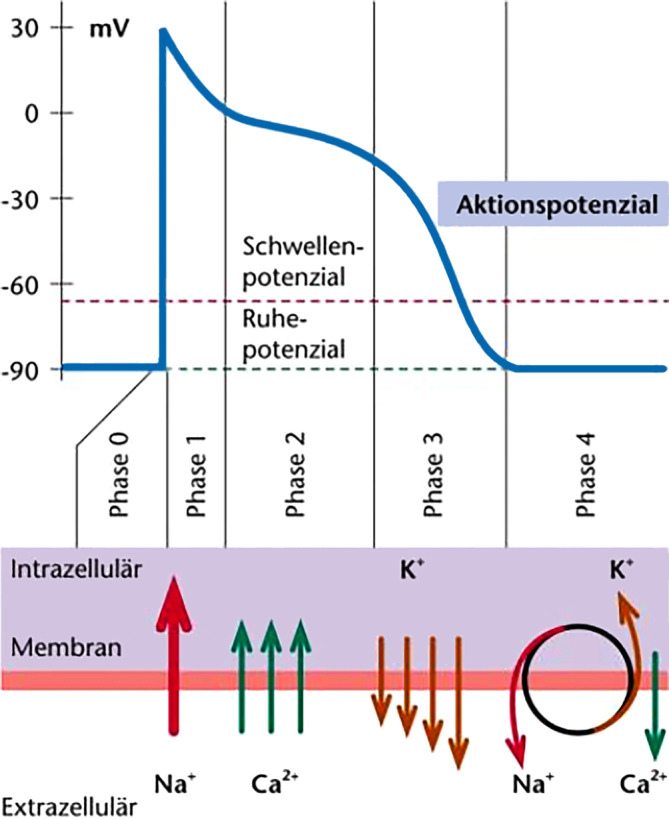
Kardiales Aktionspotenzial. Schematische Darstellung der verschiedenen Phasen des kardialen Aktionspotenzials, der beteiligten Ionenkanäle und Ionenströme. (Aus [51]. Mit freundl. Genehmigung © Elsevier GmbH, Deutschland. Alle Rechte vorbehalten)

Kenntnisse des kardialen Aktionspotenzials sowie der daran beteiligten Ionenkanäle sind elementar für ein grundlegendes Verständnis der verschiedenen kardialen Kanalopathien (Abb. 1). In Ruhe wird das Zellmembranpotenzial im Arbeitsmyokard (−90 mV) durch K^+^-Kanäle (I_K1_) aufrechterhalten. Das Aktionspotenzial einer Herzmuskelzelle wird durch die Depolarisation ihrer Nachbarzellen ausgelöst, mit denen sie durch leitende Gap Junctions verbunden ist. Die Depolarisation führt zur Öffnung spannungsabhängiger Na^+^-Kanäle, die bei etwa −60 mV aktiviert werden. Der initiale schnelle Na^+^-Einstrom (I_Na_) führt zur unmittelbaren Depolarisation der Zellmembran. Die Na^+^-Kanäle werden innerhalb von Millisekunden inaktiviert. Durch die initiale Depolarisation werden jedoch die einwärts gleichrichtenden K^+^-Kanäle (I_K1_) verschlossen und spannungsabhängige L‑Typ-Ca^2+^-Kanäle geöffnet. Die Unterbindung des repolarisierenden K^+^-Ausstroms sowie der Ca^2+^-Einstrom halten die Depolarisation dann für einige Hundert Millisekunden aufrecht (Plateau). Erst die verzögerte Aktivierung von K^+^-Kanälen („delayed rectifier K^+^ channels“, „rapid“ [I_Kr_] und „slow“ [I_Ks_]) und die verzögerte Inaktivierung von Ca^2+^-Kanälen leiten dann die Repolarisation ein. Dies führt zur Öffnung der K^+^-Kanäle (I_K1_), die für das Ruhepotenzial verantwortlich sind.

Kenntnisse des kardialen Aktionspotenzials sind elementar für ein Grundverständnis von Kanalopathien

Die Depolarisation der Kardiomyozyten führt über die Aktivierung membranständiger spannungsabhängiger L‑Typ-Ca^2+^-Kanäle zum Einstrom von Ca^2+^ in das Zytoplasma. Ca^2+^ bindet an eng benachbarte Ryanodin-Rezeptoren (Ryanodin-Rezeptor 2 [RyR2]) in der Membran des sarkoplasmatischen Retikulums (SR), das als intrazellulärer Ca^2+^-Speicher fungiert. RyR2-Kanäle öffnen sich nach Ca^2+^-Bindung, sodass große Mengen Ca^2+^ aus dem SR in das Zytoplasma freigesetzt werden (Ca^2+^-induzierte Ca^2+^-Freisetzung). Es kommt zu einem schnellen Anstieg der intrazellulären Ca^2+^-Konzentration. Dieser Anstieg der zytosolischen Ca^2+^-Konzentration löst über eine Konformationsänderung des Troponin-Tropomyosin-Komplexes die Muskelkontraktion aus. Bei der Repolarisation werden die spannungsabhängigen Ca^2+^-Kanäle geschlossen und Ca^2+^ wird aktiv zurück in das SR (über die Ca^2+^-ATPase des sarko-/endoplasmatischen Retikulums [SERCA2a]) bzw. passiv aus der Zelle transportiert (Na^+^/Ca^2+^-Austauscher [NCX]). Dies führt zur Dissoziation von Ca^2+^ und Troponin, sodass Tropomyosin die Myosinbindungsstellen am Aktin wieder blockieren kann und der Muskel sich entspannt.

## Long-QT-Syndrom

### Prävalenz und Diagnosekriterien

Die Diagnose eines Long-QT-Syndroms (LQTS) wird nach Ausschluss sekundärer Ursachen bei

- Nachweis einer QTc-Zeit ≥ 480 ms in wiederholten 12-Kanal-EKG-Ableitungen oder
- einem LQTS-Risiko-Score > 3 oder
- Nachweis einer LQTS-definierenden Mutation unabhängig von der QTc-Zeit

gestellt [1]. Die Diagnose LQTS sollte weiterhin bei wiederholten QTc-Zeiten ≥ 460 und < 480 ms und einer stattgehabten, potenziell rhythmogenen Synkope in Betracht gezogen werden [1]. Klinisch ist das LQTS durch eine pathologisch verlängerte QT-Zeit und das Auftreten polymorpher ventrikulärer Tachykardien (VT), typischerweise Torsade-de-pointes-Tachykardien, gekennzeichnet. Die Prävalenz der LQTS liegt bei 1:2500 und das Durchschnittsalter bei Erstdiagnose beträgt etwa 14 Jahre [1, 2].

Bei LQTS existiert ein geschlechtsspezifischer Unterschied bzgl. des Risikos für den plötzlichen Herztod

Bei der Mehrzahl der LQTS-Patienten findet sich im Ruhe-EKG eine pathologische QTc-Zeit-Verlängerung. Eine nichtdiagnostische QTc-Zeit-Verlängerung schließt allerdings das Vorliegen eines LQTS („concealed LQTS“) nicht aus [1, 3]. Der LQTS-Risiko-Score („Schwartz-Score“) ermittelt auf Basis von

- EKG-Parametern (QTc-Zeit, T‑Wellen-Morphologie),
- Synkopenanamnese und
- etwaiger familiärer Belastung durch LQTS bzw. plötzlichen Herztod

eine Wahrscheinlichkeit für das Vorliegen eines LQTS [4]. Trotz aller Limitationen ist dieser Score insbesondere bei Patienten mit einer unauffälligen QTc-Zeit in Ruhe wertvoll.

### Genetik und Pathophysiologie des LQTS

Bisher wurden bei Patienten mit kongenitalem LQTS pathogene und wahrscheinlich pathogene Varianten in mindestens 17 Genen identifiziert [5]. Mutationen in den drei Genen KCNQ1 (LQT1), KCNH2 (LQT2) und SCN5A (LQT3) machen insgesamt 80–90 % aller hereditären LQTS-Fälle aus [1, 5]. Bei schätzungsweise 10–20 % der Patienten mit einer sicheren klinischen Diagnose eines LQTS kann keine pathogene Mutation nachgewiesen werden.

**Abb. 2:**
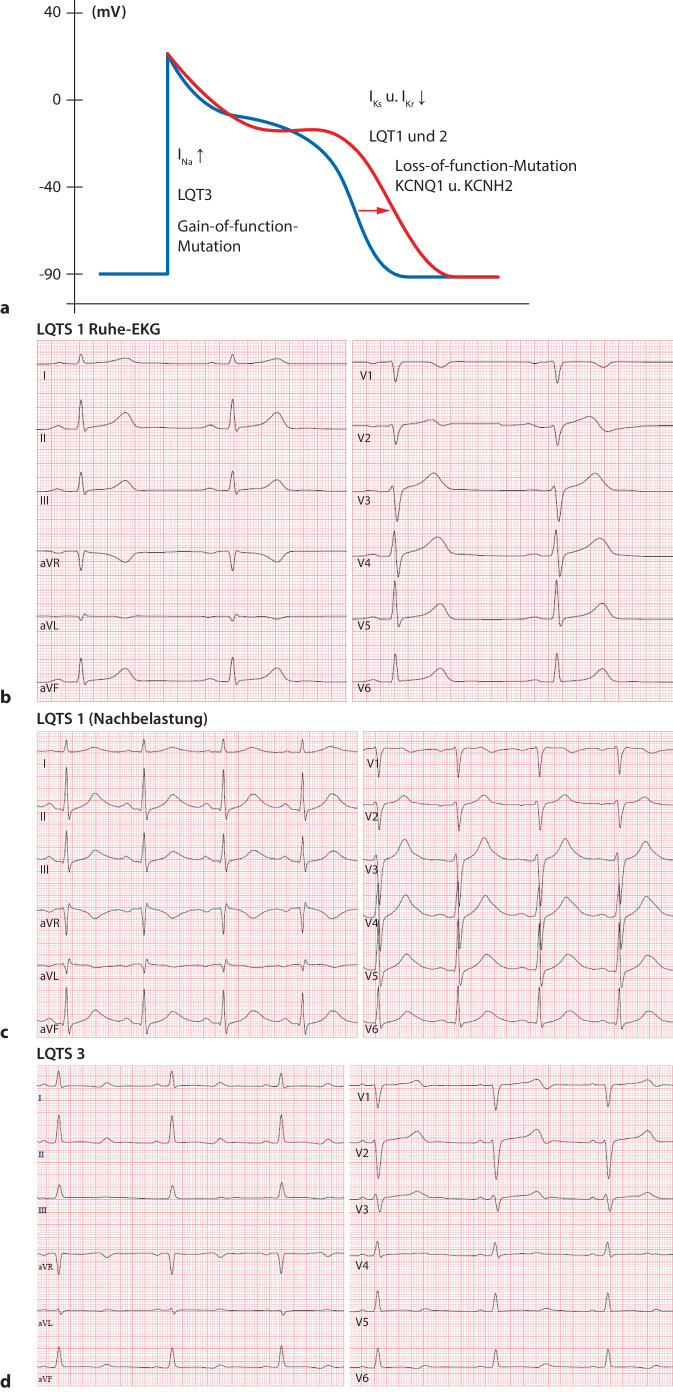
Long-QT-Syndrom. **a** Schematische Darstellung des veränderten Aktionspotenzials (*rote Potenzialkurve*) sowie der jeweils genotypspezifischen Alteration der Ionenströme bei Vorliegen eines Long-QT-Syndroms (LQT1–3). **b** 12-Kanal-EKG (50 mm/s). Ruhe-EKG bei LQT1. QTc-Zeit 430 ms. **c** 12-Kanal-EKG (50 mm/s). EKG-Registrierung während der Nachbelastungsphase nach der Fahrradergometrie bei LQT1. Signifikante Verlängerung der QTc-Zeit auf 540 ms. **d** 12-Kanal-EKG (50 mm/s). LQT3 mit langer isoelektrischer ST-Strecke und niedriger Amplitude der T‑Welle. QTc-Zeit 500 ms. *EKG* Elektrokardiogramm, *I*_*Kr*_*, I*_*Ks*_*, I*_*Na*_ verschiedene Ionenkanäle (Beschreibung siehe Haupttext), *LQT1, 2, 3* Long-QT-Syndrom Typ 1, 2, 3

Die den Long-QT-Syndromen Typ 1 (LQT1) und Typ 2 (LQT2) zugrunde liegenden Loss-of-function-Mutationen haben dysfunktionale „delayed rectifier K^+^ channels“ (I_Ks_, I_Kr_) zur Folge. Die konsekutiv reduzierten K^+^-Ströme führen zu einer Verlängerung der Plateauphase des kardialen Aktionspotenzials und damit zu einer verlängerten QT-Zeit. Die dem Long-QT-Syndrom Typ 3 (LQT3) zugrunde liegende Gain-of-function-Mutation von Na^+^-Kanälen resultiert in einer gestörten Inaktivierung und konsekutiv in einem verstärkten späten bzw. über das Aktionspotenzial persistierenden Na^+^-Einstrom (später Na^+^-Strom, „late I_Na_“). Dies verlängert die Repolarisationsphase und damit die Dauer des Aktionspotenzials (Abb. 2a).

### Risikostratifikation

Die individuelle Einschätzung des Arrhythmierisikos ist infolge der variablen Penetranz und einer starken Variation des klinischen Phänotyps anspruchsvoll. Das Alter, die jeweils zugrunde liegende Genmutation, das Geschlecht und hormonelle Veränderungen im Laufe des Lebens haben dabei Einfluss auf das Arrhythmierisiko eines LQTS-Patienten.

Im Kindesalter ist das Risiko ventrikulärer Arrhythmien bei Jungen signifikant höher als bei Mädchen [6]. Nach der Pubertät zeigt sich jedoch bei Frauen mit LQTS ein höheres Arrhythmierisiko als bei Männern [7]. Diese Veränderungen des Arrhythmierisikos sind durch Geschlechtshormoneffekte auf die kardialen Ionenkanäle bedingt [8]. In diesem Kontext muss auch das erhöhte Arrhythmierisiko in der Schwangerschaft (LQT3) bzw. postpartal (LQT1 und 2) verstanden werden.

Längere QTc-Zeiten korrelieren mit einem erhöhten Arrhythmierisiko

Längere QTc-Zeiten korrelieren ferner mit einem erhöhten Arrhythmierisiko: In Patientengruppen mit einem QTc-Intervall von ≤ 439 ms, 440–469 ms, 470–499 ms, 500–549 ms oder ≥ 550 ms betrug das entsprechende Risiko für schwerwiegende Arrhythmien in einem Beobachtungszeitraum von 22 Jahren 0 %, 2 %, 4 %, 12 % bzw. 19 % [7]. Der „1-2‑3 LQTS-risk calculator“ kann bei der Risikostratifikation eines Patienten mit angeborenem LQTS hilfreich sein [9].

### Therapiemaßnahmen

Ein wesentlicher Bestandteil der Therapie von Patienten mit LQTS besteht in der Vermeidung QT-Zeit-verlängernder Medikamente und in der Therapie von Elektrolytstörungen. Darüber hinaus sollten genotypspezifische Arrhythmie-Trigger vermieden werden: Infolge der verminderten Aktivität von I_Ks_ kommt es bei LQT1 unter adrenerger Aktivierung zu keiner ausreichenden Verkürzung der Repolarisation (Abb. 2b, c). Daher sind Patienten mit LQT1 insbesondere bei intensiver körperlicher Belastung gefährdet. Leistungssport ist bei LQT1 kontraindiziert. Beim LQT2 treten Arrhythmien vermehrt in Zusammenhang mit einer plötzlichen Sympathikusaktivierung auf. Patienten mit LQT2 wird daher empfohlen, Wecker- und laute Klingeltöne zu vermeiden [10].

Die Gabe von Betablockern ist die medikamentöse Erstlinientherapie beim LQTS. Betablocker werden bei symptomatischen und asymptomatischen LQTS-Patienten mit QT-Zeit-Verlängerung empfohlen (Klasse I). Eine Betablockertherapie sollte auch bei asymptomatischen Mutationsträgern ohne QT-Zeit-Verlängerung erwogen werden (Klasse IIa; [1]). Die antiarrhythmische Wirksamkeit einer Betablockade ist vor allem bei Patienten mit LQT1 oder LQT2 belegt; beim LQT3 ist die antiarrhythmische Wirksamkeit geringer ausgeprägt, doch auch hier stellt die Gabe von Betablockern die medikamentöse Erstlinientherapie dar [11]. Die nichtselektiven Betablocker Nadolol und Propranolol scheinen selektiven Betablockern wie Metoprolol oder Bisoprolol hinsichtlich der antiarrhythmischen Wirksamkeit überlegen zu sein [1]. Im Sinne einer genotypspezifischen Therapie kommen bei LQT3 zusätzlich zur Betablockertherapie Na^+^-Kanal-Blocker, wie Mexiletin oder Ranolazin, zur Anwendung [12].

Implantierbare Kardioverter-Defibrillatoren (ICD) werden nach einem überlebten plötzlichen Herztod oder bei fortbestehender Rhythmusinstabilität (Synkopen, VT) unter Betablockertherapie (LQT1 und 2) bzw. unter Betablocker- und Mexiletintherapie (LQT3) empfohlen (Klasse I; [1]). Bei asymptomatischen Patienten mit LQTS kann bei Vorliegen von Hochrisikomarkern (Genotyp, QTc-Zeit, „1-2‑3 LQTS-risk calculator“) die primärprophylaktische ICD-Implantation (Klasse IIb) erwogen werden [1, 9].

Zusätzlich zu einer genetisch terminierten Verlängerung der QT-Zeit (siehe oben) kann es auch durch Umgebungsfaktoren oder verabreichte Medikamente zu einer pathologischen Verlängerung der QT-Zeit kommen. Sekundäre QT-Verlängerungen sind in der klinischen Praxis häufiger als kongenitale Long-QT-Syndrome und besitzen demnach hohe Relevanz. Auch bei einer sekundären bzw. induzierten QT-Verlängerung besteht häufig eine gewisse genetische Prädisposition. Nativ subklinische Veränderungen oder Polymorphismen im Bereich von Kanalproteinen führen hierbei zu einer Reduktion der Repolarisationsreserve, sodass eine medikamentös induzierte Reduktion repolarisierender K^+^-Ströme eine deutlichere QT-Verlängerung bewirkt [13]. Es wird dann von einem medikamentös induzierten LQTS („drug-induced long QT syndrome“ [diLQTS]) gesprochen. Neben einer schweren Hypokaliämie können insbesondere Medikamente aus den Klassen der

- Antiarrhythmika (K^+^-Kanal-Blocker),
- Antibiotika (Makrolide, Fluorchinolone),
- Serotoninwiederaufnahmehemmer und
- Antipsychotika

zu einer signifikanten Verlängerung der QT-Zeit führen. Wiederholte EKG-Kontrollen unter Einsatz oben genannter Medikamente sind daher von entscheidender Bedeutung.

## Short-QT-Syndrom

Das Short-QT-Syndrom ist eine sehr seltene, autosomal-dominant vererbte Erkrankung. Klinisch ist das Short-QT-Syndrom durch eine pathologisch kurze QT-Zeit und das frühzeitige Auftreten supraventrikulärer (Vorhofflimmern) und ventrikulärer Arrhythmien (Kammerflimmern [VF]) gekennzeichnet [14]. Die Wahrscheinlichkeit für das Auftreten eines plötzlichen Herztods bis zum 40. Lebensjahr liegt bei über 40 %, wobei bereits Säuglinge und Kleinkinder gefährdet sind [15]. Die Diagnose eines Short-QT-Syndroms kann bei

**Abb. 3:**
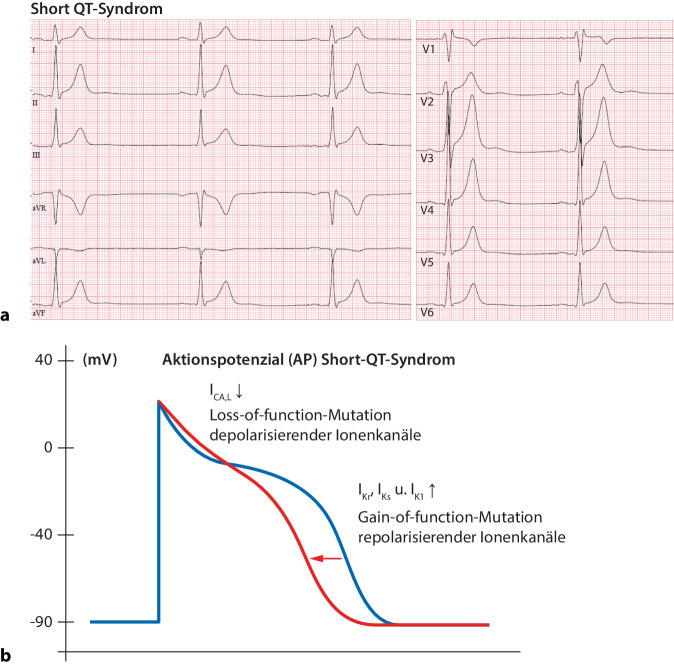
Short-QT-Syndrom. **a** 12-Kanal-EKG (50 mm/s). Spitze, zeltförmige T‑Wellen in den Extremitäten- und Brustwandableitungen. QTc-Zeit 335 ms. **b** Schematische Darstellung des veränderten Aktionspotenzials (*rote Potenzialkurve*) und der alterierten Ionenströme bei Vorliegen eines Short-QT-Syndroms. *EKG* Elektrokardiogramm, *I*_*CA,L*_*, I*_*Kr*_*, I*_*Ks*_*, I*_*K1*_ verschiedene Ionenkanäle (Beschreibung siehe Haupttext)

- Vorliegen einer QTc-Zeit ≤ 320 ms in Abwesenheit einer klinischen Symptomatik oder
- Vorliegen einer QTc-Zeit ≤ 360 ms im Kontext eines bekannten familiären Short-QT-Syndroms, eines überlebten plötzlichen Herztods oder eines Nachweises einer pathologischen Genmutation

gestellt werden (Abb. 3a; [1]). Selbstverständlich müssen Ursachen einer sekundär pathologisch kurzen QT-Zeit, wie Hyperkaliämie, Hyperkalzämie, Hyperthermie oder Azidose, ausgeschlossen werden.

Dem Short-QT-Syndrom liegt eine verkürzte Repolarisationszeit des kardialen Aktionspotenzials zugrunde. Verantwortlich hierfür sind, wie in Abb. 3b schematisch dargestellt, entweder Gain-of-function-Mutationen in Genen für repolarisierende K^+^-Kanäle (I_Kr_, I_Ks_ und I_K1_) oder Loss-of-function-Mutationen in Genen für depolarisierende Ca^2+^-Kanäle (I_CA,L_). In Abhängigkeit von der zugrunde liegenden Mutation werden die Short-QT-Syndrome 1–6 unterschieden. Das erhöhte atriale und ventrikuläre Arrhythmierisiko ist Folge der pathologisch kurzen atrialen und ventrikulären effektiven Refraktärzeiten und einer deutlichen Zunahme der transmuralen Dispersion der Repolarisation durch eine inhomogene Verkürzung der Refraktärzeiten [16].

Bei Short-QT-Syndrom sind häufig bereits Säuglinge und Kleinkinder von Arrhythmien betroffen

Klare Empfehlungen zur Risikostratifikation von Patienten mit asymptomatischem Short-QT-Syndrom liegen aktuell aufgrund der geringen Patientenzahlen nicht vor. Die primärprophylaktische ICD-Implantation wird ebenso wie die medikamentöse Therapie mit Hydrochinidin in Abwesenheit von Symptomen aktuell nicht empfohlen [1]. Bei Vorliegen eines Short-QT-Syndroms besteht jedoch nach überlebtem plötzlichem Herztod oder nach einer anhaltenden VT die Klasse-I-Indikation zur sekundärprophylaktischen ICD-Implantation [1]. Nach stattgehabter arrhythmogener Synkope sollte die ICD-Implantation (Klasse IIa) erwogen werden. Der Multikanalblocker Hydrochinidin gilt als das wirksamste medikamentöse Therapeutikum des Short-QT-Syndroms. Durch die Blockade mehrerer K^+^-Kanäle (unter anderem I_Kr_, I_Ks_ und I_K1_) kann eine Verlängerung der QT-Zeit und eine signifikante Reduktion ventrikulärer Arrhythmien erreicht werden [17, 18]. Hydrochinidin wird bei asymptomatischen Patienten mit Short-QT-Syndrom und positiver Familienanamnese für den plötzlichen Herztod sowie bei Patienten mit sekundärprophylaktischer ICD-Indikation und Ablehnung eines ICD empfohlen [1].

Das Konzept der Katheterablation ventrikulärer Extrasystolen zur Elimination von Triggern für VT und VF könnte sich zukünftig als weitere Therapieform etablieren [19].

## Brugada-Syndrom

Das BrS ist durch eine pathognomonische, schulterförmige Coved-type-ST-Strecken-Hebung in den rechts-präkordialen Ableitungen definiert und mit einem erhöhten Risiko für den plötzlichen Herztod verbunden [20, 21]. Die Prävalenz des BrS beträgt 1:2000 bis 1:5000, wobei grundsätzlich das männliche Geschlecht häufiger betroffen ist [21].

### Genetik und Pathophysiologie des Brugada-Syndroms

**Abb. 4:**
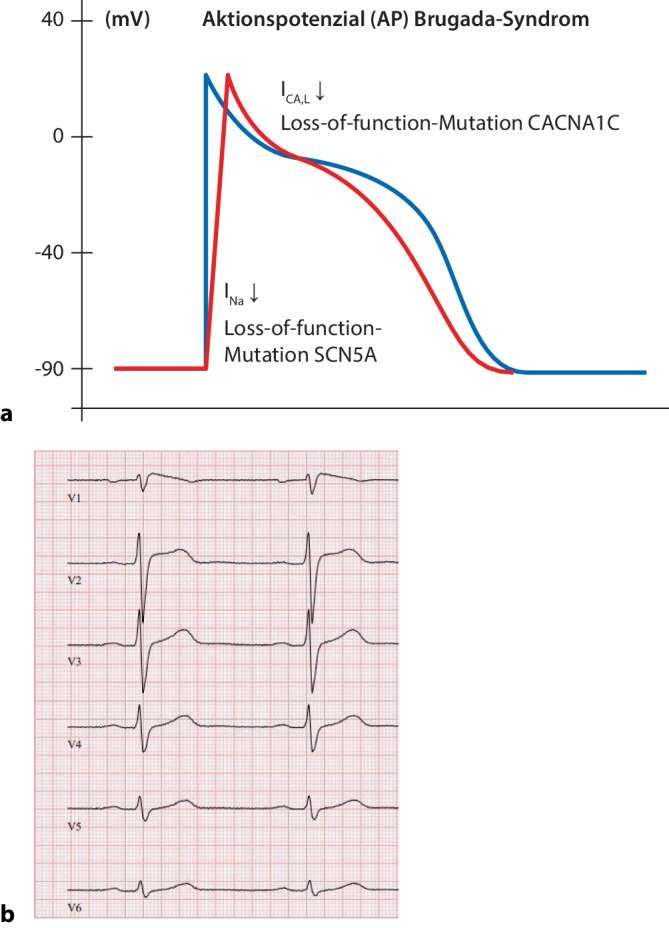
Brugada-Syndrom. **a** Schematische Darstellung des veränderten Aktionspotenzials (*rote Potenzialkurve*) mit verzögerter Depolarisation sowie der ursächlichen Alteration der Ionenströme bei Vorliegen eines Brugada-Syndroms. **b** 12-Kanal-EKG (50 mm/s). Schulterförmig gewölbte ST-Strecken-Hebung ≥ 2 mm, Kreuzung der isoelektrischen Linie durch das absteigende ST-Segment und negative, symmetrische T‑Welle in Ableitung V1. *EKG* Elektrokardiogramm, *I*_*CA,L*_*, I*_*Na*_ verschiedene Ionenkanäle (Beschreibung siehe Haupttext)

Die kardiale Depolarisation entsteht durch die Aktivierung spannungsabhängiger Na^+^-Kanäle (Isoform Na_V_1.5, SCN5A) und dauert im Allgemeinen lediglich 1–2 ms. Das BrS ist durch dysfunktionale kardiale Na^+^-Kanäle mit der Folge einer späteren Aktivierung und früheren Inaktivierung sowie durch eine verminderte Dichte dieser Na^+^-Kanäle in der Zellmembran gekennzeichnet (Abb. 4a). Dies führt zu einer Verlangsamung der Depolarisation und einer Verkürzung der Repolarisation [22].

Aktuell gelten ausschließlich *SCN5A*-Genvarianten als definiert krankheitsverursachend [23]. Allerdings findet sich nur bei etwa 20 % der Patienten mit BrS eine *SCN5A-*Variante [24]. Genomweite Assoziationsstudien legen nahe, dass das Vorhandensein mehrerer Einzelnukleotidpolymorphismen für die Mehrzahl der BrS-Fälle verantwortlich sein könnte [25]. Wichtig ist, dass dieses polygenetische Risiko das familiär gehäufte Auftreten des BrS ohne Nachweis einer einzelnen pathogenen Genvariante erklären kann.

Die funktionellen Auswirkungen der dysfunktionalen Na^+^-Kanäle kommen im Bereich des rechtsventrikulären Ausflusstrakts („right ventricular outflow tract“ [RVOT]) besonders zum Tragen. Sowohl die gestörte Depolarisation (Leitungsverzögerung) als auch die gestörte Repolarisation (endo- und epikardiale Heterogenität) können das Auftreten der BrS-typischen EKG-Phänomene und die Initiation von VT und VF erklären [16, 26, 27]. Neben diesen rein elektrischen Phänomenen werden vermehrt strukturelle Veränderungen zur Erklärung des BrS beschrieben: Histopathologische Studien zeigen, dass BrS-Patienten eine erhöhte Kollagen- und Fibrosebildung im Bereich des anterioren RVOT aufweisen und dass diese histopathologischen Veränderungen anatomisch mit epikardialen Arealen niedriger Signalamplitude („low voltage“) und fraktionierter Signale korrelieren [28, 29]. Insgesamt scheint das Konzept der verminderten Leitungsreserve des RVOT, das elektrische und strukturelle Störungen vereint, ein vielversprechender Ansatz zur Erklärung des BrS zu sein [26].

### Typ-1- und Typ-2-EKG-Veränderungen

Die möglichen BrS-assoziierten EKG-Veränderungen können intraindividuell im Tagesverlauf starken Schwankungen unterliegen, sodass ein Individuum zu unterschiedlichen Zeitpunkten sowohl ein unauffälliges Ruhe-EKG als auch ein diagnostisches Typ-1-Brugada-EKG zeigen kann. Das Typ-1-Brugada-EKG („coved type“) ist durch eine schulterförmig gewölbte ST-Strecken-Hebung ≥ 2 mm, die Kreuzung der isoelektrischen Linie durch das absteigende ST-Segment und eine negative, symmetrische T‑Welle in mindestens einer rechts-präkordialen Ableitung (V1–V3) gekennzeichnet (Abb. 4b). Das Typ-2-Brugada-EKG („saddleback type“) kennzeichnen eine konkave, sattelartige ST-Strecken-Hebung ≥ 2 mm sowie eine positive T‑Welle in mindestens einer rechts-präkordialen Ableitung (V1–V3; [30]). Ein spontan aufgetretenes Typ-1-Brugada-EKG erlaubt die eindeutige Diagnose eines BrS (siehe unten), wohingegen das Typ-2-Brugada-EKG nur einen Hinweis auf ein BrS darstellt. Durch die Applikation von Klasse-I-Antiarrhythmika, typischerweise Ajmalin, kann bei EKG-morphologischen Hinweisen auf ein BrS die Induzierbarkeit eines Typ-1-Brugada-EKGs überprüft werden. Klasse-I-Antiarrhythmika blockieren zusätzlich die bei BrS dysfunktionalen Na^+^-Kanäle (Loss-of-function-Mutation) und führen so zu einer Aggravation der EKG-Auffälligkeiten. In diesem Fall wird von einem medikamenteninduzierten Typ-1-Brugada-EKG gesprochen.

### Diagnosekriterien des Brugada-Syndroms

Ein spontan aufgetretenes Typ-1-Brugada-EKG erlaubt die eindeutige Diagnose eines BrS. Des Weiteren wird nach überlebtem plötzlichem Herztod im Falle eines medikamenteninduzierten Typ-1-Brugada-EKGs oder nach Auftreten eines Typ-1-Brugada-EKGs bei Fieber die Diagnose eines BrS gestellt. Dies ist auch der Fall bei medikamenteninduziertem Typ-1-Brugada-EKG und Vorliegen von mindestens einem der folgenden Risikofaktoren:

- Arrhythmogene Synkope
- Familiäre Belastung durch BrS
- Plötzlicher Herztod bei Verwandten ersten Grades < 45 Jahren

Die alleinige Induktion eines Typ-1-Brugada-EKGs bei asymptomatischen Patienten mit unauffälliger Familienanamnese erlaubt nicht die Diagnose eines BrS. Hier wird von einem „Brugada-Muster“ gesprochen [1].

### Klinische Manifestation des Brugada-Syndroms

Das BrS kann zum Auftreten von polymorphen VT und VF führen. Folglich sind Synkopen und der plötzliche Herztod klinische Manifestationen. Arrhythmien treten in der Regel in Phasen eines erhöhten Vagotonus, typischerweise im Schlaf bzw. in Ruhephasen, auf und werden meist durch kurz angekoppelte ventrikuläre Extrasystolen ausgelöst. Alkoholkonsum, schwere Mahlzeiten und febrile Temperaturen erhöhen ebenfalls das Arrhythmierisiko [31–33]. Das BrS wird in der Regel im Erwachsenenalter klinisch manifest, wobei das seltener betroffene weibliche Geschlecht einen zweigipfligen Verlauf mit Auftreten von Symptomen in der Kindheit oder im fortgeschrittenen Lebensalter aufweist. Das Alter bei Auftreten eines plötzlichen Herztods liegt bei 41 ± 15 Jahren [34]. Atriale Arrhythmien treten gehäuft bei Patienten mit BrS auf; die Prävalenz von Vorhofflimmern liegt bei 10 % [35].

### Risikostratifikation

Die Risikostratifikation des BrS stellt insbesondere bei der Mehrzahl asymptomatischer Patienten eine große Herausforderung dar. Es herrscht Konsens, dass ein spontan aufgetretenes Typ-1-Brugada-EKG mit einem erhöhten Arrhythmierisiko verbunden ist [1]. In einer jüngst veröffentlichten Studie wurde für Patienten mit spontanem Typ-1-Brugada-EKG eine um den Faktor 13 erhöhte Rate lebensbedrohlicher Arrhythmien (0,4 %/Jahr) gegenüber Patienten mit medikamenteninduziertem Typ-1-Brugada-EKG (0,03 %/Jahr) beschrieben [36]. Wichtig ist, dass aufgrund des fluktuierenden Charakters der pathognomonischen EKG-Veränderungen das Auftreten eines spontanen Typ-1-Brugada-EKGs in dieser Studie mittels wiederholter Ruhe- und 12-Kanal-Langzeit-EKGs bei Patienten mit initial nichtdiagnostischem Ruhe-EKG überprüft wurde. Wiederholte unauffällige Ruhe- und insbesondere 12-Kanal-Langzeit-EKG-Untersuchungen haben damit unserer Ansicht nach eine zentrale Bedeutung bei der Identifikation von Patienten mit einem sehr geringen Arrhythmierisiko.

Ein spontan aufgetretenes Typ-1-Brugada-EKG ist mit einem erhöhten Arrhythmierisiko verbunden

Der Stellenwert einer programmierten Ventrikelstimulation ist nicht abschließend geklärt [1, 36, 37]. Unter Umständen kann die Induzierbarkeit einer anhalten VT bei Patienten mit spontanem Typ-1-Brugada-EKG eine besonders gefährdete Subgruppe identifizieren [36]. Mehrere multivariate Risikoscores wurden zur Risikostratifikation asymptomatischer Patienten mit BrS entwickelt. Der zuletzt veröffentlichte Predicting-Arrhythmic-evenT(PAT)-Score schätzt anhand von 15 gewichteten Risikofaktoren das Risiko für lebensbedrohliche Arrhythmien ein [37]. Ein PAT-Score ≥ 10 Punkte konnte das Auftreten einer lebensbedrohlichen Herzrhythmusstörung mit einer Sensitivität von 96 % und einer Spezifität von 89 % vorhersagen [37]. Hier müssen jedoch die Ergebnisse prospektiver Untersuchungen abgewartet werden.

### Therapiemaßnahmen

Allgemeinmaßnahmen zu beachten und umzusetzen, ist für die Behandlung des BrS von zentraler Bedeutung. Eine Vielzahl an Medikamenten, unter anderem Klasse-Ic-Antiarrhythmika, Betablocker, verschiedene Psychopharmaka und Narkotika, können proarrhythmisch wirken und sollten unbedingt vermieden werden. Unter https://www.brugadadrugs.org werden kontraindizierte ebenso wie risikoarm zu verabreichende Medikamente gelistet. Exzessiver Alkoholkonsum ist aufgrund der proarrhythmischen Wirkung ebenfalls zu vermeiden. Fieber stellt bei Patienten mit BrS einen Notfall dar und sollte umgehend medikamentös gesenkt werden.

Bei symptomatischen Patienten mit BrS (überlebter plötzlicher Herztod, anhaltende VT) ist die sekundärprophylaktische ICD-Implantation klar indiziert (Klasse I; [1]). Infolge eines hohen Arrhythmierisikos wird die ICD-Implantation ferner bei BrS-Patienten mit potenziell arrhythmogener Synkope und spontanem Typ-1-Brugada-EKG empfohlen (Klasse IIa; [1]). Aufgrund des sehr niedrigen Arrhythmierisikos asymptomatischer BrS-Patienten ohne spontanes Typ-1-EKG wird eine primärprophylaktische ICD-Implantation bei diesen Patienten aktuell nicht befürwortet.

Bei Auftreten eines elektrischen Sturms (≥ 3 VT/VF-Episoden in 24 h) werden β‑Sympathomimetika (Isoproterenol), die durch die Erhöhung des Na^+^-Einstroms das kardiale Membranpotenzial stabilisieren, sowie I_to_-Blocker wie Hydrochinidin eingesetzt [1]. Daten weisen darauf hin, dass die epikardiale substratbasierte Ablationstherapie als effektive rhythmusstabilisierende Therapie bei rezidivierenden ICD-Therapien eingesetzt werden kann [38, 39]. Die Katheterablation asymptomatischer Patienten mit BrS wird allerdings nicht empfohlen (Klasse III; [1]).

## Katecholaminerge polymorphe ventrikuläre Tachykardie

Die CPVT ist durch die Induktion bidirektionaler oder polymorpher VT bei adrenerger Aktivierung in Abwesenheit einer strukturellen Herzerkrankung oder myokardialen Ischämie gekennzeichnet [40, 41]. Die Prävalenz der CPVT beträgt 1:10.000 [30]. Kennzeichen der CPVT sind die frühe klinische Manifestation, häufig im ersten Lebensjahrzehnt, und ein deutlich erhöhtes Risiko für den plötzlichen Herztod. Bei etwa 30 % der Patienten kommt es im Vorfeld der Diagnosestellung zu lebensbedrohlichen Arrhythmien [42, 43]. Belastungsassoziierte Synkopen oder komplexe ventrikuläre Arrhythmien während körperlicher Belastung bei ansonsten gesunden Personen sollten den Verdacht auf eine CPVT lenken [44, 45].

### Pathophysiologie

Späte Nachdepolarisationen aufgrund einer Mutation in Genen, die für Proteine des Ryanodin-Rezeptor-Komplexes codieren, sind der Arrhythmie-Trigger der CPVT [46–48]. Unter adrenerger Aktivierung kommt es dabei über den mutierten Ryanodin-Rezeptor-Komplex zu einer pathologisch gesteigerten diastolischen Ca^2+^-Freisetzung aus dem SR (SR-Ca^2+^-Leck). Die freigesetzten Ca^2+^-Ionen werden aus dem Zytoplasma über den NCX vermehrt im Austausch gegen Na^+^ nach extrazellulär befördert. Da ein Ca^2+^-Ion gegen 3 Na^+^-Ionen ausgetauscht wird, kommt es zu einem Nettoeinwärtsstrom positiver Ladungsträger und damit zu einer Nachdepolarisation. Dabei ist das Purkinje-Netzwerk wesentlich für die Initiierung und Aufrechterhaltung der bidirektionalen VT verantwortlich [46–48].

### Diagnosekriterien

**Abb. 5:**
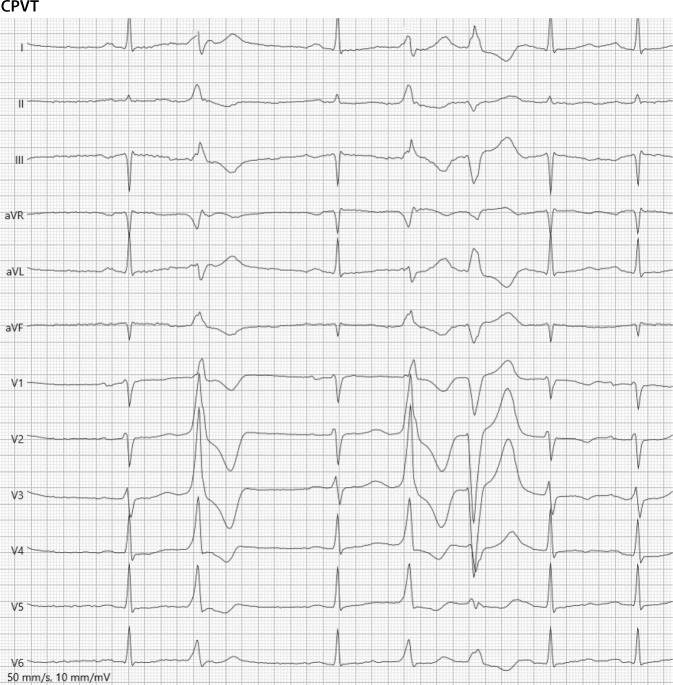
Katecholaminerge polymorphe ventrikuläre Tachykardie. 12-Kanal-EKG (50 mm/s). Fahrradergometrie. Ventrikuläres Couplet mit wechselnder QRS-Achse

Eine belastungsassoziiert auftretende bidirektionale VT, gekennzeichnet durch das typische Muster einer von Schlag zu Schlag wechselnden QRS-Achse in mindestens einer EKG-Ableitung, erlaubt die Diagnose einer CPVT (Abb. 5; [40, 41]). Ferner muss bei Nachweis einer krankheitsverursachenden Mutation (RyR2, Calsequestrin‑2 [CASQ2]) die Diagnose einer CPVT gestellt werden [1].

### Therapieempfehlungen

Patienten, die an einer CPVT leiden, müssen auf stärkere sportliche Aktivitäten verzichten. Die Therapie mit Betablockern ohne intrinsische sympathomimetische Aktivität (Nadolol, Propranolol) in der maximal tolerierten Dosierung wird bei allen Patienten und auch bei asymptomatischen Mutationsträgern empfohlen [1, 42]. Im Falle einer unzureichenden Rhythmusstabilität unter Betablockertherapie wird die additive Therapie mit Flecainid aufgrund dessen zusätzlicher RyR2-blockierender Wirkung empfohlen [1]. Bei ausgewählten Patienten, die eine Intoleranz gegenüber einer Betablockertherapie zeigen, besteht ferner die Option einer alleinigen medikamentösen Therapie mit Flecainid [49]. Die kardiale sympathische Denervierung ist eine zusätzliche Therapieoption bei fortbestehender Rhythmusinstabilität trotz optimaler medikamentöser Therapie (Klasse-IIa-Empfehlung).

Die Indikation zur ICD-Implantation wird bei CPVT kritisch gestellt

Die Indikation zur ICD-Implantation wird insgesamt kritisch gestellt. Eine Klasse-I-Empfehlung zur ICD-Implantation besteht nur nach überlebtem plötzlichem Herztod [1]. Bei allen anderen Patienten sollte die ICD-Implantation erst bei Fortbestehen von Synkopen oder dokumentierten VT unter kombinierter Therapie mit Betablockern und Flecainid kritisch abgewogen werden (Klasse-IIa-Empfehlung; [1]). Die kritische Indikationsstellung liegt darin begründet, dass insbesondere inadäquate ICD-Schocks infolge der resultierenden adrenergen Aktivierung proarrhythmisch wirken können. Retrospektive Untersuchungen belegen die Gefahr der Induktion eines tödlichen elektrischen Sturms nach inadäquatem ICD-Schock bei Patienten mit CPVT [50].

## Fazit für die Praxis

- Hereditäre Arrhythmiesyndrome sind im Allgemeinen seltene Erkrankungen.
- Im Kindes‑, Jugend- und jungen Erwachsenenalter sind hereditäre Arrhythmiesyndrome allerdings eine häufige Ursache des plötzlichen Herztods.
- Durch eine medikamentöse Therapie, eine differenzierte Aufklärung über Verhaltensmaßnahmen sowie gegebenenfalls eine ICD-Implantation lassen sich Prognose und Lebensqualität der Patienten signifikant verbessern. Daher ist eine frühzeitige Diagnose von enormer Bedeutung.
- Ein hoher Stellenwert kommt der genetischen Diagnostik zu. Diese ermöglicht in bestimmten Fällen die weitere Risikostratifikation und unter Umständen eine genotypspezifische Therapie.
